# *SR-B1* knockdown suppresses breast cancer cell proliferation, migration, and invasion via the PI3K/AKT pathway

**DOI:** 10.31744/einstein_journal/2026AO1908

**Published:** 2026-04-22

**Authors:** Ming Li, Yanmei Xu, Jinwen Li, Juan Li

**Affiliations:** 1 Department of Thoracic Surgery Shandong Public Health Clinical Center Jinan China Department of Thoracic Surgery, Shandong Public Health Clinical Center, Jinan, China.; 2 Department of Pathology Shanxian Central Hospital Heze China Department of Pathology, Shanxian Central Hospital, Heze, China.; 3 Department of Clinical Laboratory The Fourth Hospital of Jinan Jinan China Department of Clinical Laboratory, The Fourth Hospital of Jinan, Jinan, China.; 4 Office of Standardized Residency Training The Fourth Hospital of Jinan Jinan China Office of Standardized Residency Training, The Fourth Hospital of Jinan, Jinan, China.; 5 Jinan Medical Association Jinan China Jinan Medical Association, Jinan, China.

**Keywords:** SR-B1, Carcinoma, ductal, breast, PI3K/AKT pathway, Breast neoplasms, Cell proliferation, Scavenger Receptors, Class B

## Abstract

**Background:**

The role of scavenger receptor class B type 1 (SR-B1) in breast cancer remains largely unknown.

**Objective:**

This study aimed to investigate the effects of *SR-B1* on breast cancer cell proliferation, migration, and invasion and to elucidate the underlying mechanisms.

**Methods:**

Two breast cancer cell lines, MDA-MB-231 and MCF-7, were used in this study. Cells were transfected with SR-B1-specific siRNA (si-SR-B1), whereas cells transfected with scrambled sequences served as controls. Cell proliferation was assessed using CCK-8 and colony formation assays. Cell migration and invasion were evaluated using wound healing and Transwell assays, respectively. Apoptosis was analyzed using flow cytometry. Western blot analysis was performed to examine activation of the PI3K/AKT signaling pathway following *SR-B1* knockdown.

**Results:**

Knockdown of *SR-B1* significantly inhibited the proliferation, migration, and invasiveness of MDA-MB-231 and MCF-7 cells (all p<0.05). Moreover, *SR-B1* knockdown promoted apoptosis in these cells. Western blot analysis revealed that the phosphorylation levels of AKT and mTOR were markedly decreased in the si-SR-B1 group compared to those in the controls. Additionally, the expression of downstream targets, including cyclin D1 and P70, was downregulated by SR-B1 silencing.

**Conclusion:**

*SR-B1* contributes to the enhanced proliferation and migration of breast cancer cells, likely through activation of the PI3K/AKT signaling pathway.

## HIGHLIGHTS

■ *SR-B1* knockdown inhibits breast cancer cell proliferation and migration.

■ Silencing SR-B1 promotes apoptosis in breast cancer cells.

■ PI3K/AKT pathway activity decreases after SR-B1 knockdown.

■ Cyclin D1 and P70 are downregulated following SR-B1 silencing.

## In Brief

Silencing SR-B1 suppresses proliferation, migration, and invasion while promoting apoptosis in MDA-MB-231 and MCF-7 cells. *SR-B1* knockdown inhibits PI3K/AKT pathway activation, reduces AKT and mTOR phosphorylation, and downregulates downstream targets.

## Graphical abstract

**Figure:**
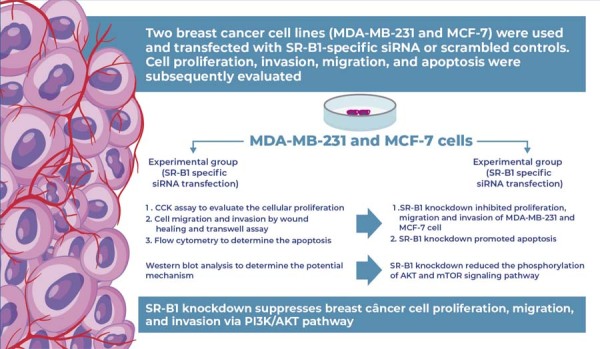

## INTRODUCTION

Breast cancer is the most common malignancy in women worldwide.^(1)^ To date, breast cancer treatment largely relies on surgery, radiotherapy, chemotherapy, hormone therapy, and targeted therapy. However, numerous patients experience treatment failure due to recurrence and metastasis.^(2,3)^

Scavenger receptor class B type 1 (SR-B1) is an integral membrane protein located in caveolae and plays a role in the hepatic uptake of cholesteryl esters. It plays a crucial role in the metabolism of high-density lipoproteins (HDL) and reverse cholesterol transport (RCT).^(4)^ Additionally, *SR-B1* functions as an oncogene and is overexpressed in several cancers, including breast cancer.^(5)^For example, Wang et al. reported that low SR-B1 protein levels are associated with more aggressive tumor phenotypes.^(6)^ Moreover, SR-B1 upregulation has been proposed as a potential biomarker for predicting the prognosis of patients with clear cell renal cell carcinoma (CCRCC).^(5)^ Nevertheless, the role of SR-B1 in the pathogenesis of breast cancer remains poorly understood. In our previous study, we found that SR-B1 was upregulated in invasive ductal breast cancer (IDCA) tissues and that its elevated expression was linked to increased tumor size and unfavorable post-treatment prognosis.^(7)^ However, the molecular mechanisms through which SR-B1 contributes to breast cancer progression remain unclear.

## OBJECTIVE

This study aimed to investigate the biological functions of SR-B1 in breast cancer cell proliferation, invasion, and apoptosis. In addition, we examined the activation of the PI3K/AKT signaling pathway following *SR-B1* knockdown, with the aim of identifying the downstream target of *SR-B1* in these processes.

## METHODS

### Cell culture

Two human breast cancer cell lines, MCF-7 and MDA-MB-231, were used. The cells were obtained from the Department of Pathology, Shandong University School of Medicine, and cultured in RPMI 1640 medium (Gibco, Carlsbad, CA, USA) supplemented with 10% fetal bovine serum (FBS; Beyotime, Shanghai, China) at 37°C in a humidified atmosphere with 5% CO_2_. Upon reaching 80-90% confluence, the cells were passaged by trypsinization. Both the MCF-7 and MDA-MB-231 cells were authenticated by KeyGEN Biotech (Nanjing, China) using short tandem repeat (STR) analysis before purchase. All experiments were conducted using mycoplasma-free cells.

### Small interfering RNA (siRNA) transfection

Cells in the logarithmic phase were seeded onto six-well plates, and transfected with siRNA against *SR-B1* (si-SR-B1; 5’-CCT GAA TGA TGT GTC TCT GCA-3’) purchased from RiboBio (Guangzhou, China) using Lipofectamine 2000 (Invitrogen, CA, USA). Cells transfected with scrambled siRNA were used as controls. Transfection efficiency was evaluated using real-time PCR and Western blotting.

### CCK-8 and colony formation assays

Approximately 24 h after transfection, the cells (1×10^2^) were subjected to a CCK-8 assay using a commercial kit (RiboBiotech, Guangzhou, China). Cellular proliferation in each group was measured every 24 h according to the manufacturer’s instructions. For the colony formation assay, cells (1 × 10^2^) were seeded into 60 mm culture dishes 24 h after transfection. After 14 days of culture, the colonies were fixed and stained with 1% crystal violet (Solarbio, Beijing, China). The number of colonies was counted and normalized for further analysis.

### Wound healing assay

After siRNA transfection, a uniform scratch was made across the cell monolayer when the cells reached 100% confluence. Detached cells and debris were removed by washing with PBS, and the culture medium was replaced with medium containing 2% FBS. Images of the wound area were captured every 12 h, and the migration distance was quantified after 24 h using ImageJ software (version 1.53).

### Transwell assay

Cells (1×10^2^) suspended in serum-free medium were seeded into the upper chamber of a Transwell insert (Thermo Fisher, Lenexa, KS, USA). The lower chamber was filled with 600µL of DMEM containing 10% FBS. After incubation at 37°C in a CO_2_ incubator overnight, the cells that had invaded and adhered to the lower surface of the membrane were fixed and stained with 1% crystal violet. The number of invading cells in five randomly selected fields was counted under a microscope.

### Flow cytometry

After transfection, apoptosis was induced by serum starvation for 24 h. Harvested cells were centrifuged for 5 min and resuspended in PBS. Subsequently, approximately 1 × 10^2^ cells were stained with 5μL Annexin V-FITC and 10μL PI in the dark at room temperature. Apoptosis was analyzed using a flow cytometer (NovoCyte, Agilent, Palo Alto, CA, USA), and data were processed using FlowJo software.

### Real-time PCR

Total RNA was extracted from cells using TRIzol reagent (Thermo Fisher, Lenexa, KS, USA). Complementary DNA (cDNA) was synthesized using a commercial kit (Qiagen). Amplification was performed using SYBR Green on a Real-Time PCR system (Bio-Rad, Hercules, CA, USA). The specific primers for *SR-B1* were as follows: 5’-CAT CAA GCA GCA GGT CCT TA-3’; 5’-CGT CAA AGA AGT AGA CGG AGAG-3’. The amplification conditions were as follows: 95°C for 5 min, followed by 40 cycles of 95°C for 15 s and 60°C for 1 min. Relative gene expression levels were calculated using the 2^CT^ method.

### Western blot analysis

Total protein was extracted from both cell lines and its concentration was determined using a BCA assay kit (Kangwei Biotech, China). Equal amounts of protein were separated by SDS-PAGE and transferred onto PVDF membranes (Millipore, Billerica, MA, USA). The membranes were blocked with 5% skim milk and incubated overnight at 4°C with primary antibodies against SR-B1 (1:1000; Novus, USA), Bcl-2 (1:1000, PTG, USA), Bax (1:1000, PTG, USA), Caspase-3 (1:1000, PTG, USA), PTEN (1:1000, PTG, USA), cyclin D1 (1:1000, PTG, USA), and P70 (1:1000, PTG, USA), AKT (1:1000, CST, USA), p-AKT (1:1000, CST, USA), mTOR (1:1000, CST, USA) and p-mTOR (1:1000, CST, USA). After washing, the membranes were incubated with horseradish peroxidase-conjugated goat anti-rabbit IgG (1:5000; ZSGB-BIO, Beijing, China). Membranes probed with GAPDH were used as the internal standard. All bands were visualized using Image Lab software (Bio-Rad, Hercules, CA, USA), and band intensities were quantified using ImageJ software.

### Statistical analysis

Data are presented as mean ± standard deviation. Statistical analyses were performed with SPSS 19.0 software. All experiments were performed at least in triplicates. Student’s t-test was used for comparisons between two groups, whereas one-way analysis of variance (ANOVA) was used for comparisons among multiple groups. Statistical significance was considered in the presence of a p-value of less than 0.05.

## RESULTS

### *SR-B1* knockdown suppressed the proliferation of breast cancer cells

**Figure 1:**
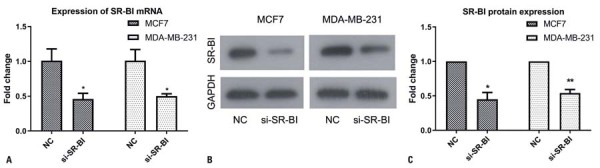
Transfection efficiency of *SR-B1* in breast cancer cells assessed by real-time PCR and western blot analysis. (A) MCF-7 and MDA-MB-231 cells were transfected with si-SR-B1 or the negative control (NC), and *SR-B1* mRNA levels were significantly lower in the si-SR-B1 group compared with their values in the NC. (B, C) SR-B1 protein levels were significantly lower in MDA-MB-231 and MCF-7 cells following *SR-B1* knockdown compared with the Control Group

**Figure 2:**
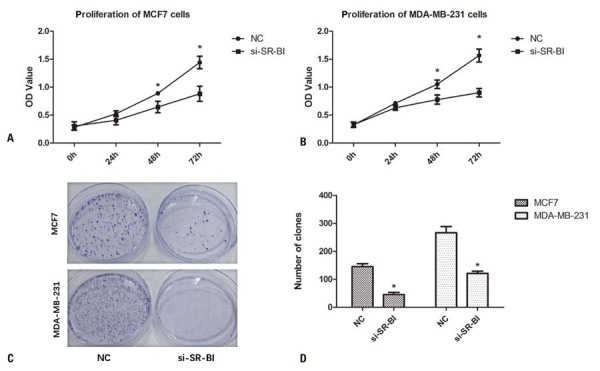
*SR-B1* knockdown inhibited proliferation and colony formation in MCF7 and MDA-MB-231 cells. (A, B) Proliferation of MCF7 and MDA-MB-231 cells based on the CCK-8 method. (C, D) Colony formation was markedly reduced in MCF-7 and MDA-MB-231 cells following *SR-B1* knockdown

The transfection efficiency of the siRNA was validated using real-time PCR and Western blot analysis (Figure 1A-C). *SR-B1* knockdown significantly reduced the proliferation of MDA-MB-231 and MCF-7 cells at 48 and 72 h compared to that in the Control Group (p<0.05, Figure 2A). Similarly, the knockdown of *SR-B1* markedly decreased colony formation compared to the Control Group (p<0.05, Figure 2B and C).

*p<0.05 *versus*; **p<0.01 *versus* NC.

*p<0.05 *versus* NC.

### *SR-B1* knockdown inhibited the migration and invasion of breast cancer cells

**Figure 3:**
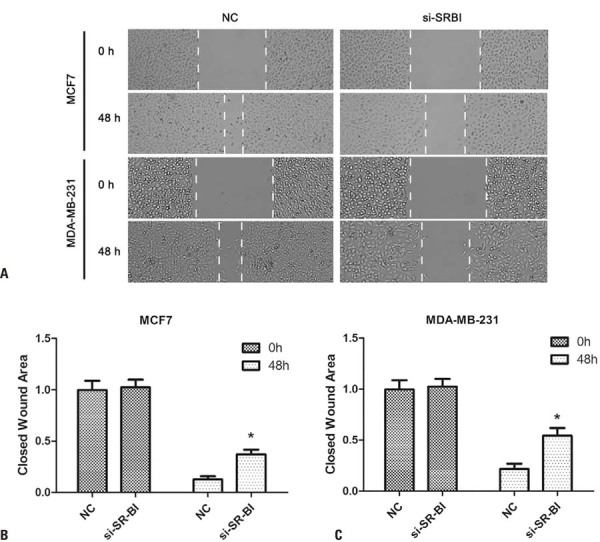
*SR-B1* knockdown inhibited the invasion of breast cancer cells. (A) Wound healing of MCF7 and MDA-MB-231 cells at 0 h and 48 h. (B, C) Following *SR-B1* knockdown, cancer cell invasion was significantly increased compared with the NC group at 48 h

**Figure 4:**
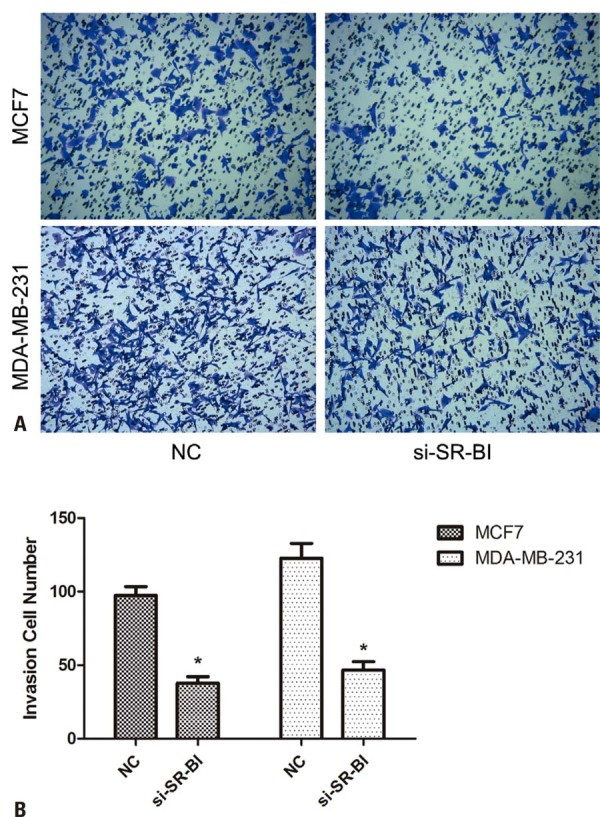
*SR-B1* knockdown could inhibit the invasion of MCF7 and MDA-MB-231 cells. (A) Transwell assay performed on MCF7 and MDA-MB-231 cells. (B) The invasion decreased following *SR-B1* knockdown

Compared with the Control Group, *SR-B1* knockdown significantly suppressed the migration of MCF-7 and MDA-MB-231 cells at 48 h (p<0.05, Figure 3A and B). Transwell assays showed that *SR-B1* silencing markedly inhibited the invasiveness of both cell lines (p<0.05, Figure 4A and B).

*p<0.05 *versus* NC.

*p<0.05 *versus* NC.

### *SR-B1* knockdown promoted the apoptosis of breast cancer cells

**Figure 5:**
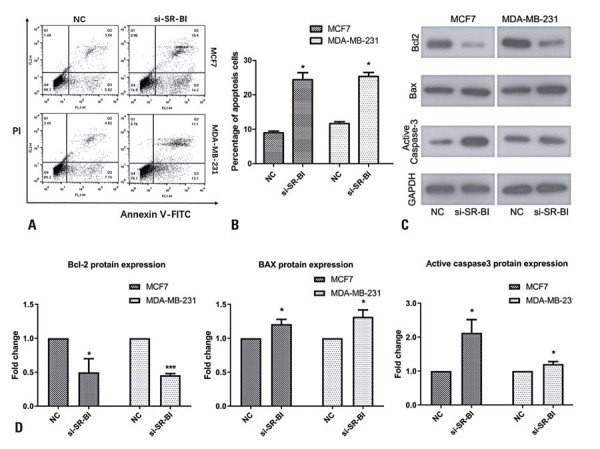
Effects of *SR-B1* knockdown on cellular apoptosis. (A, B) Flow cytometry indicated a significant increase in cancer cell apoptosis after *SR-B1* knockdown. (C, D) After *SR-B1* knockdown, Bcl-2 expression was significantly down-regulated, and the expression of Bax and active caspase-3 was significantly up-regulated

Compared with the Control Group, *SR-B1* knockdown significantly increased apoptosis in MCF-7 and MDA-MB-231 cells (p<0.05, Figure 5A and B). In addition, silencing of *SR-B1* markedly upregulated the expression of pro-apoptotic proteins, including Bax and active Caspase-3, while downregulating the anti-apoptotic protein Bcl-2, compared to the Control Group (Figure 5C and D).

*p<0.05 *versus* NC; ***p<0.001 *versus* NC.

### *SR-B1* knockdown regulated the PI3K/AKT signaling pathway

**Figure 6:**
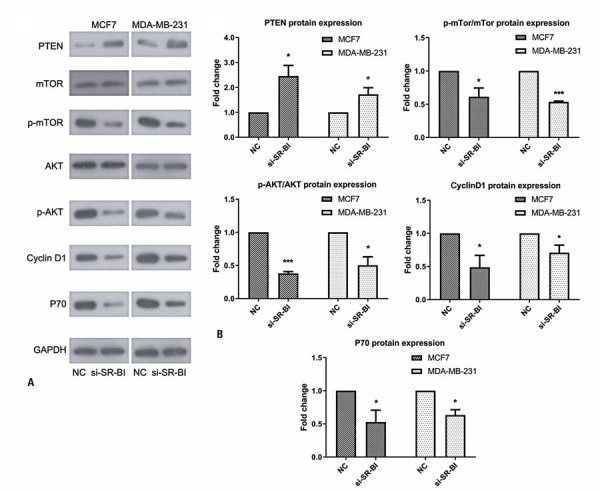
After *SR-B1* knockdown, the phosphorylation of AKT and mTOR showed a significant decrease, and the expression of cyclin D1 and P70 was also downregulated (all p<0.05). (A, B) Western blotting analysis and the semi-quantitative results

Knockdown of *SR-B1* inhibits activation of the PI3K/AKT signaling pathway. The expression of PTEN, a key molecular switch in the PI3K/AKT signaling pathway, was significantly upregulated in the si-SR-B1 group compared to the Control Group. The phosphorylation of AKT and mTOR was markedly reduced following *SR-B1* knockdown (p<0.05). Additionally, the expression of downstream target genes, including *cyclin D1* and *P70*, was downregulated following *SR-B1* knockdown (p<0.05; Figure 6A and B). These results indicate that *SR-B1* knockdown regulates the PI3K/AKT signaling pathway, which may be related to the regulation of cellular proliferation and migration in breast cancer.

*p<0.05 *versus* NC; ***p<0.001 *versus* NC.

## DISCUSSION

*SR-B1* is aberrantly expressed in several cancers, including prostate, nasopharyngeal, ovarian epithelial, and breast cancers.^(8)^ In a mouse xenograft model, *SR-B1* expression was markedly upregulated in castration-resistant prostate cancer cells.^(9)^ Our previous study demonstrated that SR-B1 was significantly overexpressed in IDCA tissues and correlated with tumor size and poor prognosis.^(7)^ In this study, we investigated the effects of *SR-B1* on the proliferation, invasion, and apoptosis of breast cancer cells. Our data showed that *SR-B1* knockdown inhibited the migration, invasion, and proliferation of breast cancer cells, and promoted apoptosis.

The Bcl-2 protein family plays a central role in the mitochondria-mediated intrinsic apoptotic pathway,^(10)^ and its activity is primarily regulated by the phosphorylation of apoptotic proteins.^(11)^ In the mitochondrial apoptotic pathway, the balance between Bcl-2 and Bax influences the activation of downstream Caspase-3,^(12)^ which regulates cell survival and apoptosis. Activated Caspase-3 can be cleaved at Asp34, and Bcl-2 can be processed into a fragment exhibiting pro-apoptotic activity similar to that of Bax, collectively promoting apoptosis.^(13)^ In our study, *SR-B1* silencing significantly downregulated Bcl-2 expression, accompanied by upregulation of Bax and activation of Caspase-3. These results suggest that *SR-B1* modulates breast cancer cell proliferation, metastasis, and apoptosis, as evidenced by decreased migration and proliferation, and increased apoptosis following *SR-B1* knockdown.

SR-B1 regulates HDL metabolism and activates downstream signaling molecules including Src kinase, PI3K, and AKT.^(14)^ Previous studies have shown that HDL/SR-B1-mediated lipid transport activates Src kinase and the PI3K/AKT signaling pathway^(15)^ and that cholesterol transport via HDL-SR-B1 plays a crucial role in cancer cell proliferation and invasion.^(16)^ The PI3K-AKT signaling pathway is closely associated with cancer pathogenesis by regulating cell proliferation and the cell cycle.^(17)^ Additionally, it modulates angiogenesis, which is a key step in metastasis.^(18)^Accordingly, targeting the PI3K/AKT pathway has been explored as a therapeutic strategy, because it can be aberrantly activated by various mechanisms, including PTEN, AKT, and mTOR signaling.^(19)^ As a major downstream effector of AKT, mammalian target of rapamycin (mTOR) promotes cancer progression by interacting with several proteins.^(20)^ In line with these findings, our data demonstrated that *SR-B1* knockdown inhibited the activation of the PI3K/AKT signaling pathway. Specifically, the phosphorylation of AKT and mTOR was significantly reduced after *SR-B1* knockdown, and the expression of downstream target genes, including cyclin D1 and P70, was significantly decreased. These results indicated a close relationship between *SR-B1* and PI3K/AKT, which may play a key role in the pathogenesis of breast cancer.

This study had several limitations. Although we have demonstrated that *SR-B1* regulates cancer cell proliferation, invasion, and apoptosis, the precise underlying mechanisms remain unclear. This study was primarily designed to investigate the effects of *SR-B1* knockdown, and rescue experiments were not included in the original experimental design. Nevertheless, our current results consistently demonstrate that *SR-B1* knockdown suppresses the proliferation, migration, and invasion of breast cancer cells, accompanied by reduced AKT/mTOR phosphorylation. The pathogenesis of breast cancer is multifactorial and involves genetic mutations, activation of oncogenes, and loss of imbalance in tumor suppressor genes. Further in-depth studies are required to investigate the role of *SR-B1* in the molecular and biological mechanisms of breast cancer.

## CONCLUSION

*SR-B1* promotes the proliferation, invasion, and migration of MDA-MB-231 and MCF-7 cells. *SR-B1* knockdown induces apoptosis and inhibits cell proliferation, migration, and invasion by suppressing the PI3K/AKT signaling pathway.
